# Multi-walled carbon nanotube instillation impairs pulmonary function in C57BL/6 mice

**DOI:** 10.1186/1743-8977-8-24

**Published:** 2011-08-18

**Authors:** Xiaojia Wang, Pranita Katwa, Ramakrishna Podila, Pengyu Chen, Pu Chun Ke, Apparao M Rao, Dianne M Walters, Christopher J Wingard, Jared M Brown

**Affiliations:** 1 Department of Pharmacology & Toxicology, Brody School of Medicine, East Carolina University, Greenville, North Carolina, USA; 2 Department of Physics and Astronomy, Clemson University, Clemson, South Carolina, USA; 3 Department of Physiology, Brody School of Medicine, East Carolina University, Greenville, North Carolina, USA

**Keywords:** MWCNT, pulmonary fibrosis, pulmonary function, Ccl3, Ccl11, Mmp13, IL-33, Flexivent, Raman spectroscopy

## Abstract

**Background:**

Multi-walled carbon nanotubes (MWCNTs) are widely used in many disciplines due to their unique physical and chemical properties. Therefore, some concerns about the possible human health and environmental impacts of manufactured MWCNTs are rising. We hypothesized that instillation of MWCNTs impairs pulmonary function in C57BL/6 mice due to development of lung inflammation and fibrosis.

**Methods:**

MWCNTs were administered to C57BL/6 mice by oropharyngeal aspiration (1, 2, and 4 mg/kg) and we assessed lung inflammation and fibrosis by inflammatory cell infiltration, collagen content, and histological assessment. Pulmonary function was assessed using a FlexiVent system and levels of Ccl3, Ccl11, Mmp13 and IL-33 were measured by RT-PCR and ELISA.

**Results:**

Mice administered MWCNTs exhibited increased inflammatory cell infiltration, collagen deposition and granuloma formation in lung tissue, which correlated with impaired pulmonary function as assessed by increased resistance, tissue damping, and decreased lung compliance. Pulmonary exposure to MWCNTs induced an inflammatory signature marked by cytokine (IL-33), chemokine (Ccl3 and Ccl11), and protease production (Mmp13) that promoted the inflammatory and fibrotic changes observed within the lung.

**Conclusions:**

These results further highlight the potential adverse health effects that may occur following MWCNT exposure and therefore we suggest these materials may pose a significant risk leading to impaired lung function following environmental and occupational exposures.

## Background

The use of nanomaterials has been prominent in recent years due to their diverse properties and applications. Carbon nanotubes, in particular, possess the potential for numerous modifications and display unique physical and chemical properties, making them the ideal choice for product development in technological and biomedical industries [1]. Despite increasing use of carbon nanotubes, there is limited research on the potentially detrimental effects to human health and safety. In existing studies of carbon nanotube exposure in animal models, multi-walled carbon nanotubes (MWCNT) have been shown to potentiate allergic, inflammatory and fibrotic pulmonary responses. These effects have been associated with significant increases in pro-inflammatory cytokines such as IL-5, IL-6, IL-33 and others [2]. Inoue *et al*. determined that MWCNT exposure augments pulmonary inflammation induced by initial LPS activation of cytokines [3]. Post-exposure periods as early as 7 days have demonstrated increased collagen accumulation, development of granulomas and fibrosis in murine lungs exposed to MWCNT levels comparable to human exposure levels [4]. Ma-Hock *et al*. showed similar data with MWCNT inhalation in rats 90 days post-exposure [5]. Development of fibrotic tissue in lungs of mice exposed to MWCNT has been shown to correlate with increased macrophage and epithelial cell mediated production of platelet derived growth factor AA (PGDF-AA), a major mediator of pulmonary fibrosis [6]. In addition to inducing adverse pulmonary effects, exposure to MWCNTs has also been shown to augment pre-existing allergic responses. Ryman-Rassmusen *et al*. demonstrated that mice exposed to MWCNT following ovalbumin sensitization developed significantly increased airway fibrosis and lung inflammation compared to mice that were not treated with MWCNTs but challenged with ovalbumin [7]. This suggests that pre-existing allergic conditions may predispose individuals to adverse effects of MWCNTs [7]. While these data validate the potential adverse effects, few studies have been conducted to confer these findings in the context of pulmonary physiology. A recent study by North *et al*. demonstrates altered pulmonary function with exposure to particulate matter [8]. It was shown that in animals challenged with ovalbumin, exposure to ambient air particles augments airway resistance and hyperresponsiveness [8]. While MWCNTs are known to augment pre-existing allergic conditions, they are also able to independently produce adverse pulmonary responses. However, the effects of MWCNTs on pulmonary function have yet to be determined.

Pulmonary function testing is a valuable tool to evaluate phenotypic characteristics of mouse respiratory disease that might be caused by nanoparticle exposure. Hamilton *et al*. have demonstrated that BALB/c mice exposed to carbon nanoparticles had increased airway hyperresponsiveness as measured by changes in PenH using barometric whole body plethysmography [9]. This study suggests potential for nanoparticles to influence pulmonary function; however, alterations in specific lung function parameters have not been reported. The FlexiVent provides a method to directly measure pulmonary function via the use of preprogrammed ventilator and system-specific maneuvers such as forced oscillation technique (FOT) [10]. FOT measures the respiratory impedance (Z) which is considered a detailed measurement of pulmonary mechanics [11]. FOT is an administration of small pressure oscillations at the airway opening using an external generator while simultaneously recording the oscillatory pressure and flow signals [12,13]. Respiratory impedance (Z) is a complex quantification represented by two components: respiratory resistance (Rrs) and reactance (Xrs). Rrs is derived from airways and lung tissue resistance, whereas Xrs is determined by the inertial (I) and elastic (E) properties of the respiratory system [14]. This technique provides a means of distinguishing central airways from peripheral airways and lung parenchyma.

In the current study, we tested the hypothesis that instillation of MWCNTs impairs pulmonary function in C57BL/6 mice due to development of lung inflammation and fibrosis. As will be shown, mice instilled with MWCNTs exhibited increased inflammatory cell infiltration, collagen deposition, and granuloma formation that led to deteriorating pulmonary function 30 days following instillation. Pulmonary exposure of MWCNTs induces an inflammatory signature marked by cytokine (IL-33), chemokine (Ccl3 and Ccl11), and protease (Mmp13) production which collectively promote inflammatory and fibrotic changes within the lung.

## Results

### MWCNT characterization

**Figure 1:**
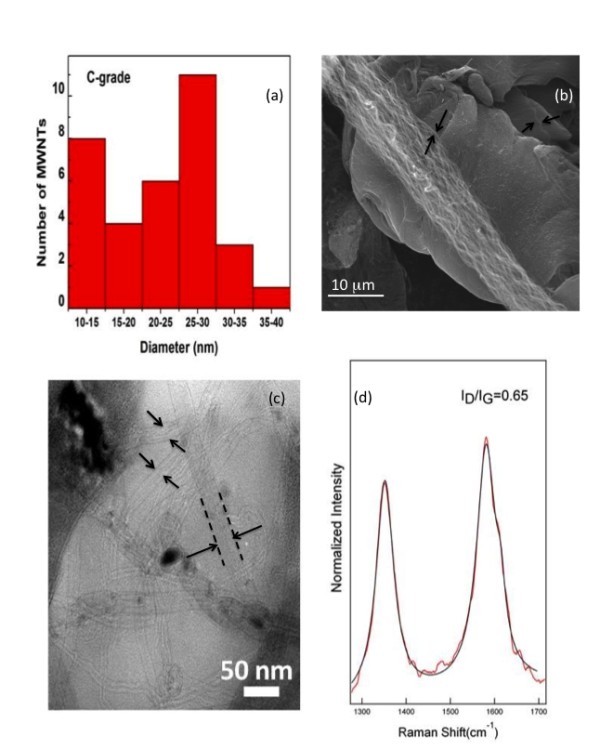
**Characterization of MWCNT in Dry Powder Form**. Characterization of multi-walled carbon nanotubes was determined using electron microscopy and Raman spectroscopy. (A & C) The diameter of the MWNCTs was determined by measuring individual nanotubes visualized by TEM. (B) This SEM image shows a bundle of MWCNTs, with individual nanotubes indicated by arrows. The length of individual nanotubes was determined to be several μm long using SEM. (D) The Raman spectrum of MWCNT obtained using 514.5 nm laser excitation. The presence of strong disorder band (I_D_/I_G _peak ratio) suggests the existence of structural defects as determined by Raman spectroscopy.

**Figure 2:**
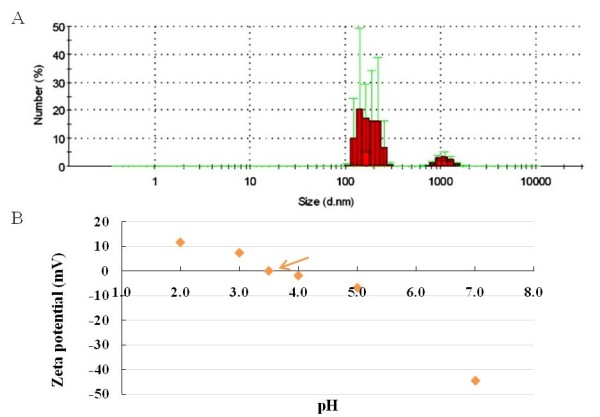
**Characterization of MWCNT in Suspension**. The size and charge characteristics of the MWCNTs were determined in a suspension of 10% surfactant saline. (A) The size of MWCNT bundles in suspension was determined by dynamic light scattering. (B) The zeta potential and isoelectric point (indicated by the arrow) were determined for the MWCNTs suspended in 10% surfactant saline solution.

Our electron microscopy studies revealed that the MWCNTs used in this study were several microns long with a bi-modal diameter distribution exhibiting peaks at ~12.5 and 25 nm (Figures 1A, B &1C). In MWCNT Raman spectra, the presence of strong disorder band ~1350 cm^-1 ^indicates the defective nature of the nanotubes. Specifically, the ratio (*R = I_D_/I_G_*) between the intensity of disorder band (D-band) and graphite-like band (G-band) was found to be 0.65 (Figure 1D). The elemental analysis indicated presence of Fe catalyst in MWCNT samples (Additional File 1: Figure S1 & S2). Further, the TGA studies showed that the MWCNT samples posses ~ 5 wt% of Fe catalyst (Additional File 1: Figure S1 & S2). The surface area of the MWCNTs was determined to be 113.103 m^2^/g, based on the BET equation. The pore volume of the MWCNTs, defined as the ratio of the MWCNTs' air volume to their total volume, was determined to be 0.688 cm^3^/g utilizing the BJH method. The hydrodynamic sizes of the MWCNT suspension displayed two peaks, a major one at 200 ± 50 nm and another at 1,000 ± 150 nm (Figure 2A). The larger sized peak was caused by the bundling of the nanotubes through hydrophobic interaction and pi-stacking. The MWCNT suspension displayed a zeta potential of -44.6 mV, suggesting a very stable colloidal state of the nanomaterial. The IEP of the MWCNT suspension was measured to be pH = 3.5 (Figure 2B).

### MWCNTs Induce Pulmonary Inflammation and Granuloma Formation in C57BL/6 Mice

**Figure 3:**
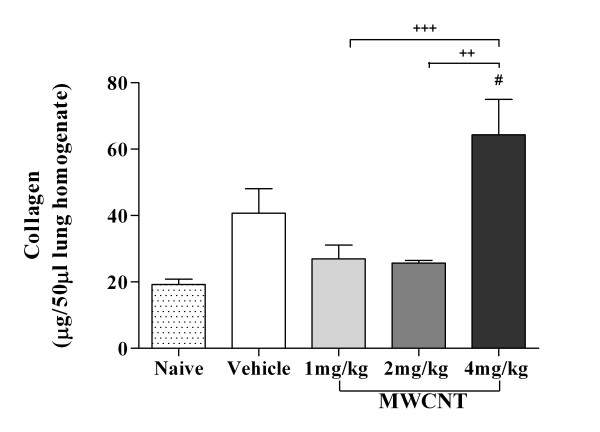
**Increased collagen content in lung tissue of MWCNT exposed C57BL/6 mice**. Collagen content was determined by lung tissue harvested from mice 30 days post-exposure to vehicle control (10% surfactant in saline) or MWCNTs (1, 2, or 4 mg/kg). Mice exposed to the high dose MWCNT (4 mg/kg) displayed significant increase in collagen levels compared to naïve mice. All values are expressed as mean ± SEM (n = 6-11). *p < 0.05 compared to naïve mice and ^+ ^*p *< 0.05 compared between two groups. No significant differences were found between naïve and vehicle treated groups. In addition, differences between vehicle and treatment groups were not significant.

**Figure 4:**
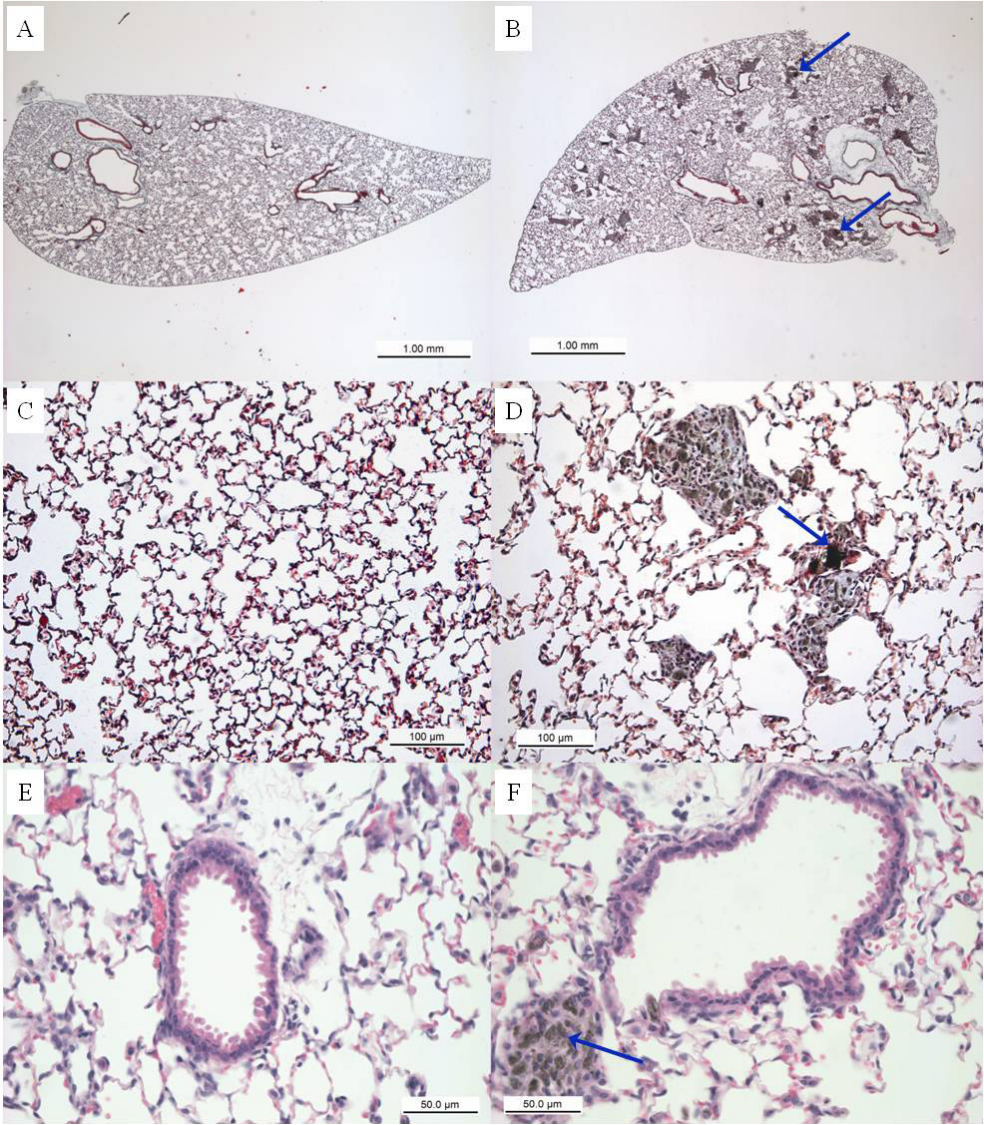
**Histopathology of lungs exposed to MWCNT displays granulomatous and fibrotic tissue at 30 days post-exposure**. Mice instilled with (A) 10% saline in surfactant vehicle control display normal lung morphology while mice instilled with (B) 4 mg/kg MWCNTs exhibit widely dispersed deposition of MWCNT aggregates within lung tissue. Masson's trichrome staining shows collagen rich granulomas and surrounding fibrotic tissue (blue) in lungs of mice exposed to (D) MWCNTs, but not (C) vehicle control. H&E staining demonstrates granulomatous peribronchioloar foci in lungs of mice exposed to MWCNT (F) but not vehicle (E). Agglomerates of MWCNT within granulomas are indicated by arrows. Images are representative of 4 mice per group with original magnifications of 25 × (A-B), 200 × (C-D) and 400 × (E-F).

To investigate the inflammatory effects of MWCNT exposure that may be associated with function changes, C57BL/6 mice treated with 10% saline/surfactant or increasing doses of MWCNTs underwent bronchoalveolar lavage (BAL) 30 days post-exposure. Differential cell counts obtained from BAL demonstrated persistent inflammation with pulmonary infiltration of multiple cell types (Table 1). Statistically significant elevations in macrophage, epithelial cell, and neutrophil numbers were observed in animals exposed to the highest dose MWCNT (4 mg/kg), along with an overall increase in total cell recruitment (*p *< 0.001). Exposure to both the 1 and 2 mg/kg MWCNT treatments displayed significant increases in epithelial and neutrophil cell counts (*p *< 0.05) compared to mice treated with vehicle. Increases in eosinophil numbers were observed with MWCNT exposure, but did not reach significance compared to control mice. Histological evaluation of lung sections confirmed the altered inflammatory cell profile and additionally revealed increased collagen deposition as well as changes in lung morphology following MWCNT aspiration. Quantification of collagen content within lung tissue demonstrated a significant increase in MWCNT (4 mg/kg) exposed mice compared to naïve mice (Figure 3). However, no statistically significant difference was found between vehicle and MWCNT treated mice, nor was there a statistical difference between naïve and vehicle treated mice. Oro-pharyngeal aspiration of MWCNTs resulted in wide distribution of granulomatous foci containing MWCNT agglomerates and MWCNT laden macrophages throughout the lung parenchyma (Figure 4B) as well as peribronchiolar and perivascular regions (Figure 4B, D &4F). In contrast, mice exposed to vehicle control did not display any inflammation or granulomas (Figure 4A, C, &4E).

**Table 1:** Effect of MWCNT instillation on pulmonary cell populations in C57BL/6 mice

Treatment	Macrophages (×10^3^)	Epithelial Cells (×10^3^)	Neutrophils (×10^3^)	Eosinophils (×10^3^)	Lymphocytes (×10^3^)	Total Cells (×10^3^)
Vehicle	113.71 ± 11.51	22.60 ± 3.80	0.18 ± 0.09	0.25 ± 0.14	0.05 ± 0.05	136.77 ± 11.68
1 mg/kg MWCNT	247.4 ± 20.77	22.0 ± 5.39^Ŧ^	0.50 ± 0.25^Ŧ^	0.66 ± 0.35	2.55 ± 1.90	273.17 ± 21.58
2 mg/kg MWCNT	230.75 ± 52.41	18.85 ± 1.78^Ŧ^	2.35 ± 1.24^Ŧ^	1.55 ± 1.03	0.10 ± 0.10	253.60 ± 54.36.
4 mg/kg MWCNT	331.62 ± 61.92**	59. 54 ± 6.34**	16.17 ± 3.90**	2.87 ± 1.67	1.24 ± 0.67	441.28 ± 68.13**

Values are mean ± SEM. N = 6/group; *p < 0.01 vs vehicle; **p < 0.001 vs vehicle, ^Ŧ^p < 0.05 vs 4 mg/kg MWCNT

### Instillation of MWCNTs in C57BL/6 Mice Elicits Dose-Dependent Changes in Pulmonary Function

**Figure 5:**
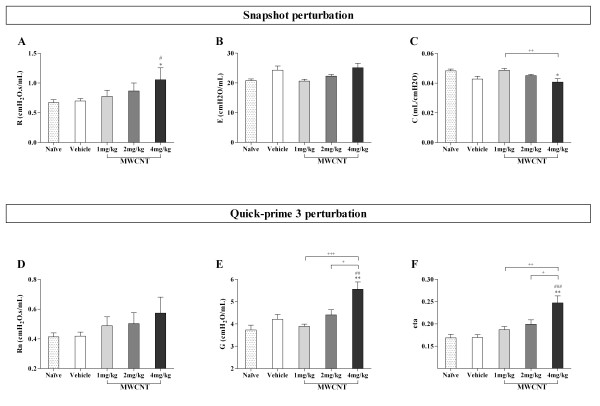
**Impaired pulmonary function as determined by Snapshot and Quick-prime 3 perturbation after MWCNT instillation**. The Snapshot and Quick-prime3 perturbations were performed in tracheotomized C57BL/6 mice instilled with increasing doses of MWCNTs. Since Snapshot perturbation measures the lung as a single compartment, the parameters, R (A), E (B), and C (C), are indicative of the whole respiratory system including lung and chest wall. On the other hand, Quick-prime 3 perturbation measures the lung as multiple compartments. The parameters can differentiate between central airway (Rn (D)) and peripheral lung tissue (G (E) and eta (F)). The mean ± SEM of six mice per group are shown, *p < 0.05 and ** *p *< 0.01 compared with the naïve mice, ^# ^*p *< 0.05 and ^## ^*p *< 0.01 compared with the vehicle control (10% surfactant in saline) mice; ^+ ^*p *< 0.05, ^++ ^*p *< 0.01, and ^+++ ^*p *< 0.001 compared between two groups. No statistically significant differences were found between naïve and vehicle treated mice.

To determine if MWCNT exposure elicits any physiologically relevant toxicity, we examined pulmonary function in C57BL/6 mice following instillation of vehicle, 1, 2, or 4 mg/kg of MWCNTs. As shown in Figure 5, the snapshot perturbation (Figure 5A, B &5C) in C57BL/6 mice revealed a dose-dependent pulmonary response to MWCNT instillation with a statistically significant increase in R compared to vehicle treated and naïve mice (Figure 5A). Further, MWCNT instillation led to a statistically significant decrease in C as compared to the naïve mice, but this did not reach significance as compared to vehicle treated mice (Figure 5C). The quick-prime 3 perturbation (Figure 5D, E &5F) further demonstrated a moderate dose dependent pulmonary response to MWCNTs with increases in Rn, G, and eta following pulmonary instillation of 1, 2, or 4 mg/kg MWCNTs. Mice treated with 4 mg/kg MWCNTs displayed significant increases in tissue damping (G) (Figure 5E) and hysteresivity (eta) (Figure 5F) compared to both the naïve and vehicle groups. Meanwhile, both G and eta displayed a significant dose dependent response to MWCNTs.

**Figure 6:**
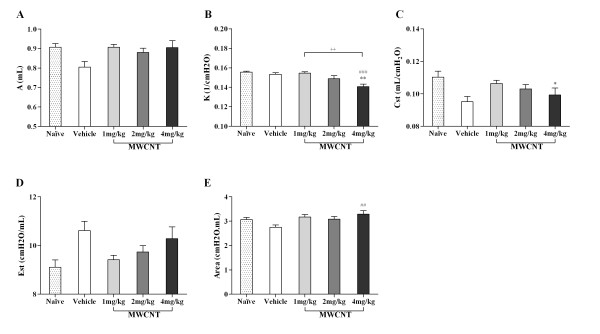
**Impaired pulmonary function as determined by PVr-P perturbation after MWCNT instillation**. The PVr-P perturbation was performed in tracheotomized C57BL/6 mice instilled with vehicle (10% surfactant in saline) or increasing doses of MWCNTs. From PV loops, perturbation parameters, (A) A, (B) K, (C) Cst, (D) Est, and (E) Area were determined. The mean ± SEM of six mice per group are shown, *p < 0.05 and ** *p *< 0.01 compared with the naïve mice; ^# ^*p *< 0.05, ^## ^*p *< 0.01, and ^### ^*p *< 0.001 compared with the vehicle control mice; ^++ ^*p *< 0.01 compared between two groups. No statistically significant differences were found between naïve and vehicle treated mice.

The PVr-P loop perturbations showed dose-dependent decreases in the deflating PV loop (K) (Figure 6B) and static compliance (Cst) (Figure 6C). Both reductions of K and Cst were statistically significant in the 4 mg/kg treated group compared to naïve mice; however, the change in Cst was not statistically different from vehicle control. As expected, Est showed the opposite trend as Cst, but differences between treatment groups were not statistically significant. In contrast, the area between the limbs of the PV loop displayed a statistically significant increase in the 4 mg/kg treated mice compared to the vehicle group (Figure 6E), but was not statistically different from naïve or other MWCNT treated mice. No statistically significant differences were found between naïve and vehicle treated mice in Figure 5 and 6.

### Ccl3, Ccl11, Mmp13 and IL-33 are Increased in the Lungs and BALF of C57BL/6 Mice instilled with MWCNTs

**Figure 7:**
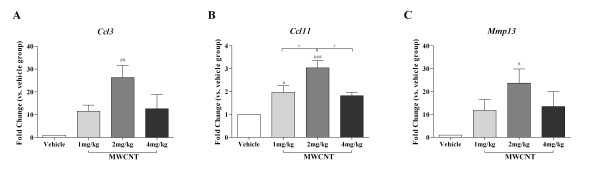
**Induction of *Ccl3, Ccl11*, and *Mmp13 *in lungs of C57BL/6 mice exposed to MWCNTs**. Real-Time PCR analysis was performed in the left lung of vehicle (10% surfactant in saline), 1 mg/kg, 2 mg/kg, and 4 mg/kg MWCNTs instilled C57BL/6 mice for genes (A) *Ccl3*, (B) *Ccl11*, and (C) *Mmp13*. The mean ± SEM of six mice per group are shown, ^# ^*p *< 0.05, ^## ^*p *< 0.01, and ^### ^*p *< 0.001 compared with the vehicle control mice; ^+ ^*p *< 0.05 compared between two groups.

To assess potential mechanisms involved in MWCNT directed pulmonary inflammation, fibrosis and alteration of lung function, we employed a mouse fibrosis PCR array to investigate the expression of 84 keys genes associated with fibrosis. Gene expression was analyzed using unlavaged lung tissue homogenates from mice exposed to vehicle or 4 mg/kg MWCNT. Messenger RNA levels of multiple fibrotic mediators that were up- or down-regulated > 2 fold in MWCNT instilled mice are shown in Table 2. To verify PCR array data on dose dependent changes in gene expression induced by MWCNT exposure, mRNA levels of *Ccl3, Ccl11*, and *Mmp13 *were analyzed in vehicle, 1, 2, and 4 mg/kg MWCNT instilled mice. As shown in Figure 7, mRNA levels of *Ccl3, Ccl11*, and *Mmp13 *were increased in all MWCNT-treated groups with a statistically significant increase observed in the 2 mg/kg MWCNT group compared to vehicle control.

**Table 2:** Lung pro-fibrotic gene expression in MWCNT instilled C57BL/6 mice compared to vehicle control

Gene	Description	p-Value	Fold Change
*Mmp13*	Matrix metallopeptidase 13	0.0253	7.92
*Ccl3*	Chemokine (C-C motif) ligand 3	0.0078	3.10
*Ccl11*	Chemokine (C-C motif) ligand 11	0.0038	2.76
*Plau*	Plasminogen activator, urokinase	0.0461	2.11
*Cebpb*	CCAAT/enhancer binding protein (C/EBP), beta	0.0243	-2.05
*Jun*	Jun oncogene	0.0366	-2.09
*Eng*	Endoglin	0.0007	-2.19
*Fasl*	Fas ligand (TNF superfamily, member 6)	0.0044	-2.20
*Smad6*	MAD homolog 6 (Drosophila)	0.0004	-2.40
*Plg*	Plasminogen	0.0290	-2.44
*Serpina1a*	Serine (or cysteine) peptidase inhibitor, clade A, member 1a	0.0312	-2.46

**Figure 8:**
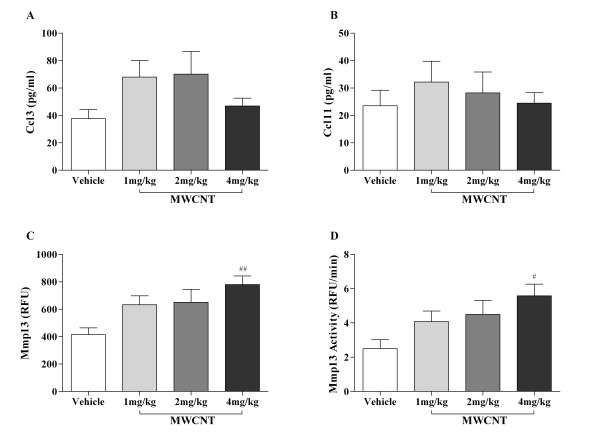
**Increased Ccl3, Ccl11, and activity of Mmp13 in BAL fluid after instillation with MWCNTs**. ELISA analysis was performed in the BAL fluid of vehicle, 1 mg/kg, 2 mg/kg, and 4 mg/kg MWCNT instilled C57BL/6 mice for chemokines (A) Ccl3 and (B) Ccl11. Collagenase activity in the BAL fluid of vehicle control (10% surfactant in saline) and MWCNT instilled mice was measured using SensoLyte^® ^Mmp13 Assay Kit. (C) The relative fluorescence units (RFUs) and (D) the reaction velocity of Mmp13 were dose-dependent increased 30 days post-MWCNT instillation. The mean ± SEM of six mice per group are shown, ^# ^*p *< 0.05 and ^## ^*p *< 0.01 compared with the vehicle control mice.

In addition to mRNA levels of *Ccl3, Ccl11*, and *Mmp13*; we assessed protein levels of chemokines, Ccl3 (Mip1α) and Ccl11 (eotaxin), and activity levels of Mmp13 in BALF of vehicle and MWCNT instilled mice. Similar to mRNA levels, both Ccl3 and Ccl11 levels were elevated in MWCNT instilled mice but did not reach significant levels when compared to vehicle treated mice (Figures 8A &8B). Thirty days post-MWCNT instillation, we observed dose-dependent increases in Mmp13 levels as well as collagenase activity in BALF from MWCNT instilled mice with statistically significant increases in the 4 mg/kg MWCNT instilled mice compared to vehicle control (Figures 8C &8D).

**Figure 9:**
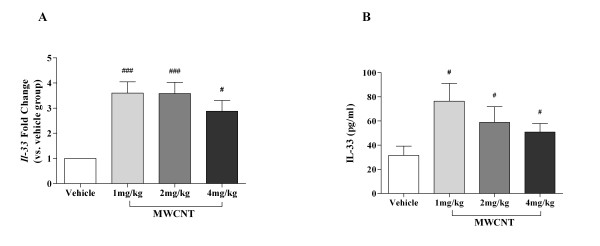
**Induction of IL-33 gene and protein expression in C57BL/6 lung tissue and BALF 30 days post-exposure to MWCNTs**. Gene expression of IL-33 (A), determined by Real-Time PCR, demonstrated an approximate three-fold increase in left lung tissue of mice instilled with MWCNTs compared to vehicle control mice. Correspondingly, ELISA protein analysis (B) of IL-33 in BALF of mice exposed to MWCNTs was also significantly increased at all doses (1, 2, and 4 mg/kg) compared to vehicle control (10% surfactant in saline). All values are expressed as mean ± SEM (n = 6-11). ^#^*p *< 0.05 compared to vehicle control mice and ^### ^*p *< 0.001 compared with the vehicle control mice.

To further investigate mechanisms involved in the inflammatory response, we identified IL-33, a novel alarmin and Th2 cytokine, as a potential mediator in MWCNT induced pulmonary inflammation. While no dose dependent change was evident, gene expression analysis of lung tissue from mice 30 days post-exposure to 1, 2, or 4 mg/kg MWCNTs demonstrated a statistically significant > 2-fold induction in *Il-33 *(Figure 9A). Similarly, assessment of IL-33 protein expression in BALF exhibited a statistically significant increase for all dose groups compared to the vehicle control (Figure 9B). There were no significant differences in *Il-33 *gene expression or protein levels between MWCNT dose groups.

## Discussion

Pulmonary toxicity of MWCNTs has been reported in both mouse and rat models [15-17]. Instillation of MWCNT into the lungs of mice and rats has been shown to induce fibrosis [18]; however, extensive evaluation of pulmonary function changes in animals instilled with MWCNTs has not been reported. In this study, we demonstrated that 30 days following MWCNT instillation, C57BL/6 mice exhibited changes in pulmonary function that were consistent with pulmonary inflammation, increased collagen deposition and granuloma formation. Additionally, increased levels of Ccl3, Ccl11, and Mmp13 were observed in C57BL/6 mice instilled with different doses of MWCNTs. Taken together, these results suggest that MWCNT exposure could lead to impaired pulmonary function due to inflammatory and fibrotic remodeling of lung tissue.

Due to the implication that MWCNTs may adversely affect human health and safety, appropriate dosing of animals was essential to evaluate the pertinence of these findings in regard to human exposure levels. Studies conducted in industrial plants indicated nanoparticle exposure levels up to 0.5 mg/m^3 ^for an 8-hour work day and 40-hour work week [19]. Additional evaluations of carbon nanotubes in manufacturing and research facilities, found airborne levels during handling to be as low as 53 μg/m^3 ^[20] and as high as 400 μg/m^3 ^[4,15]. Shvedova *et al*., report that human occupational exposure levels of 5 mg/m^3 ^over the course of an 8-hour day and 40-hour work week equate to approximately 20 μg of MWCNT aspiration in a mouse model [21]. Current proposed guidelines by the National Institute for Occupational Safety and Health (NIOSH) limits exposure to 7 μg/m^3^, the lowest detectable level of airborne CNT using the latest analytical methods [22]. The doses used in this study, 1, 2 and 4 mg/kg MWCNTs (equivalent to 27, 54, and 108 μg of MWCNT in the average 27 g mouse used in this study), were selected based on these limited exposure studies and doses previously reported in the literature for rodent studies [4]. Furthermore, the MWCNTs were dispersed in 10% surfactant in saline, a physiologically relevant medium, shown to effectively reduce aggregation of nanotubes in solution [23]. Data from this study demonstrated a persistent inflammatory response and the development of granulomatous and collagen-rich fibrotic tissue in C57BL/6 mice, similar to previously reported findings [4,7]. Post-exposure to MWCNTs at 90 days exhibited granulomatous foci similar to those found in chronic human granulomatous disorders [24]. In fact, due to their ability to produce a robust granulomatous response, the use of MWCNTs has been proposed as a novel murine model to study chronic granulomatous disease [24]. In the present study, we demonstrated that focal aggregates of MWCNTs were present at the core of the granulomas and surrounding fibrotic tissue (Figure 4D). In addition, our data indicated that intact MWCNTs do not congregate to form airway granulomas, but are dispersed throughout the lung tissue (Figure 4B) contrary to reports from previous investigators [17]. Furthermore, studies have shown that MWCNTs are not only found in the periphery of the lungs post-exposure, but have also been found to translocate to the pleural space [7]. Data from this study are consistent with previous reports that MWCNTs induce pulmonary inflammation and fibrosis, and are widely distributed through the lung following exposure. Further, we have now shown that these severe adverse pulmonary responses have a negative impact on pulmonary function.

In this study, pulmonary functional variables were evaluated in C57BL/6 mice exposed to different doses of MWCNTs using the FlexiVent system. The Snapshot perturbation was imposed to measure resistance (R), dynamic compliance (C), and elastance (E) of the whole respiratory system (including airways, lung, and chest wall). Our data showed that R was significantly increased at the highest dose of MWCNT with a concomitant declined in dynamic compliance (C). The decrease in C is consistent with findings in a bleomycin-induced model of pulmonary fibrosis in mice [10]. However, that study did not demonstrate an increase in R. This discrepancy is likely due to the fact that inflammation was attenuated by use of cyclophosphamide. Thus, in our study, the consistent elevation in numbers of inflammatory cells may have contributed to the increased R observed. Because R reflects the combined resistance contributed by both the airways and lung parenchyma, we employed the constant-phase model to distinguish between central and peripheral respiratory mechanics and to provide information about the heterogeneity of the respiratory response [12].

Tissue damping (G), which reflects parenchymal distortion, showed a dose-dependent increase in MWCNT instilled mice which was likely due to inflammatory infiltrates, as well as granuloma formation in the peribronchiolar and alveolar regions of the lung. Since these structures account for a major portion of the cross-sectional area of the lung, any obstruction in the distal airways and/or alveolar spaces could contribute to an increase in R. Furthermore, the decrease in C may suggest that the alveoli are not fully expanding as a result of alveolar volume reduction which is likely due to inflammation and/or granuloma formation. This would reduce the traction normally exerted on the conducting airways at high lung volume and thereby also contribute to changes in resistance (R). Similar to Rn, H, a measure of tissue elastance, also displayed a non-significant trend towards increased elastance with increasing doses of MWCNT (data not shown). Tissue elastance is typically elevated with fibrotic lung disease; but the patchy nature of the granulomatous/fibrotic lesions seen in this model may account for the lack of significance. However, eta (G/H) was dose-dependently increased with MWCNT instillation and was significantly elevated at the highest dose, indicating heterogeneity in the combined inflammatory and fibrotic responses to MWCNTs. On the other hand, Rn showed a trend toward dose-dependent increases that did not reach significance, suggesting that MWCNTs may have a minor effect on central airways. In support of this, we observed only minor inflammation within the central airways. However, this effect, while small with MWCNTs alone, may explain enhancement of airway responses to methacholine in models of allergic airway disease by exposure to air pollution [8] observed in other studies. Along the same line, our study showed a MWCNT-induced increase in eosinophil numbers and eotaxin levels (Ccl11) which may also contribute to MWCNT enhancement of allergic airway disease.

Static compliance (Cst) and the upper portion of the deflation PV curve (K), parameters derived from PVr-P maneuvers, declined significantly at the highest dose of MWCNTs which is typical of fibrotic lung disease. In contrast to our findings, Kamata et al. [25] reported that carbon black nanoparticles had no effect on lung compliance. This might be due to the physicochemical differences between carbon black nanoparticles and MWCNTs. A decrease in Cst was also observed in the vehicle treated mice suggesting that the administration of high doses of pulmonary surfactant reduces lung compliance.

Taken together, our pulmonary function findings suggest that MWCNT exposure results predominantly in peripheral respiratory disease as a result of combined inflammatory infiltrates and granulomatous/fibrotic parenchymal responses, reflected by a decline in pulmonary function. Consistent with other reports, we observed significant granuloma formation with fibrotic content which likely contributed to restrictive changes in pulmonary function [24]. Translocation and penetration of MWCNTs into pleural space, as previously shown, may also contribute to peripheral respiratory injury and subsequent structural changes [7,26].

To begin examining potential molecular mechanisms by which MWCNT instillation mediates impaired pulmonary function, gene and protein expression of cytokines and chemokines were examined to identify fibrotic and inflammatory responses. PCR arrays were utilized to identify changes in gene expression related to the development of fibrosis. We identified *Mmp13, Ccl3*, and *Ccl11 *as three highly upregulated gene products. Mmps can be divided by structure and substrate specificity into several subgroups including collagenases, gelatinases, stromelysins, and membrane-type (MT) Mmps [27]. Imbalanced expression of MMPs has been associated with fibrosis [28]. Mmp13, an interstitial collagenase, is considered a key activator in the cascade of proinflammatory reactions leading to pulmonary fibrosis [29]. Furthermore, activation of Mmp13 enhances the process of macrophage chemoattraction and infiltration of other inflammatory cells following tissue injury. MWCNT instilled mice exhibited a dose dependent increase in Mmp13 production and activity in BAL fluid. Additionally, Ccl3 (also named MIP-1α) is a critical macrophage chemoattractant in murine wound repair [30]. In our study, increased Ccl3 expression in the lung and Mmp13 activity in BAL fluid following MWCNT exposure are likely associated with the collagen deposition and granuloma formation in mouse lung exposed to MWCNT. Lastly, Ccl11, involved in eosinophil recruitment, was significantly increased in the lung following MWCNT instillation. This likely contributed to the increasing trend in eosinophils in the BALF seen with increasing doses of MWCNT. Consistent with previous studies [31], an influx of eosinophils into the lungs by a variety of eosinophil chemoattractants, such as Ccl11, was observed. This classic Th2 driven inflammatory response may contribute to the subsequent fibrotic outcome and change in resistance that was observed in response to MWCNT exposure.

Finally, IL-33 was examined for its potential role in inflammatory cell recruitment and Th2 immune responses following MWCNT exposure. IL-33 is a member of the IL-1 cytokine family and the only known ligand for the ST2 receptor [32]. The ST2 receptor is most highly expressed on mast cells and Th2 lymphocytes, and is known to exist in at least two isoforms; a transmembrane form and a soluble form which is cleaved after activation by IL-33 [33]. Haraldsan *et al*. describe IL-33, as a potential alarmin, or immune stimulating danger signal during trauma or infection [34,35]. Studies have confirmed IL-33 as a chemoattractant for human and murine Th2 cells [36]. Furthermore, IL-33 has recently been shown to polarize macrophages to a M2 phenotype (or alternatively activated macrophages), resulting in enhanced production of pro-inflammatory and pro-fibrotic cytokines and chemokines in Th2 driven pathologies [37]. The IL-33/ST2 axis, known to influence these Th2 cell types including mast cells, macrophages and eosinophils, has been recognized in modulating disease pathologies such as anaphylaxis and allergic asthma [38]. A study by Mangan et al. demonstrates that expression of transmembrane ST2 on Th2 cells negatively impacts both pulmonary physiologic and pathologic responses in an OVA-induced pulmonary inflammation mouse model. Changes in airways resistance measures, such as PenH, along with pathologic alterations of pulmonary cell infiltrates and cytokine profiles in ST2 knock-out mice indicate a role for IL-33 and ST2 receptor in regulating allergic inflammatory responses [39]. Further, airway hyper-reactivity following allergen challenge can be attenuated by blockade of the transmembrane ST2 receptor in BALB/c mice [40]. Thus, during injury or insult to the lung, IL-33 upregulation and activation of ST2 receptors may significantly impact Th2 driven cell types resulting inflammatory, fibrotic and physiological changes.

Our data show significant increases in IL-33 expression with MWCNT exposure compared to vehicle control, demonstrating a potential role in inflammation and fibrosis. Mechanistically, the release of chemokines (CCL3 and CCL11), cytokines (IL-33) and the activation of MMP13, help to amplify pro-inflammatory and pro-fibrotic mediators, as well as recruit macrophages, neutrophils, and eosinophils into the lung, thereby contributing not only to the pathologic inflammatory and fibrotic responses, but also the physiologic and functional changes induced by MWCNTs.

## Conclusion

In this study, we have demonstrated that MWCNT instillation results in increased immune cell infiltration, increased collagen deposition, and a granulomatous response. Further, we provide evidence of detrimental lung remodeling exhibited by impaired pulmonary function; and begin to identify mechanisms of MWCNT-induced lung remodeling through increased expression of CCL3, CCL11, MMP13 and IL-33. Given these data and the increased use of MWCNTs and other nanomaterials, we believe further investigation into the toxicity of these materials is warranted.

## Methods

### Animals

Male C57BL/6 mice were obtained from Jackson Laboratories (Bar Harbor, ME, USA) at 9 to 10 weeks of age. The average weight of the C57BL/6 mice was 27.4 ± 0.58 g. Mice were randomly assigned to five groups (6-8 mice/group) which included naïve, vehicle (10% surfactant in saline), 1 mg/kg, 2 mg/kg, or 4 mg/kg MWCNTs. Mice received a single dose of MWCNTs by oropharyngeal aspiration of 1, 2, or 4 mg/kg body weight of MWCNTs or 10% surfactant in saline as vehicle control, following administration of isoflurane anesthesia [41]. Clinical grade surfactant (Infasurf) was kindly provided by ONY company (Amherst, NY, USA). Pulmonary function testing was performed on mice 30 days post-instillation. Following pulmonary function measurements mice were euthanized for bronchoalveolar lavage and collection of lung tissue. All animal procedures were conducted in accordance with the National Institutes of Health guidelines and approved by the East Carolina University Institutional Animal Care and Use Committee. All animals were treated humanely and with regard for alleviation of suffering.

### MWCNT characterization

Commercial grade multi-walled carbon nanotubes were generously provided by NanoTechLabs, Inc (Yadkinville, NC). We performed transmission and scanning electron microscopy studies using Hitachi H-9500, S-4800 microscopes to obtain length, diameter distribution and elemental composition. Raman spectra were obtained using Ar+ ion excitation at 514.5 nm coupled with a Dilor XY triple grating monochromator equipped with thermoelectric cooled CCD. The surface area, pore volume and pore size distribution of the MWCNTs were obtained using a physisorption analyzer (Micromeritics ASAP 2010) and derived based on the Brunauer-Emmett-Teller (BET) equation [42] and the Barrett-Joyner-Halenda (BJH) method [43]. The MWCNTs were dispersed in a saline solution containing 10% surfactant at 2 μg/μl and the mixture was bath-sonicated (1510R-MTH, Branson Ultrasonics Corp.) for 45 minutes to obtain a suspension. The hydrodynamic size distribution, a parameter describing the effective diameter of a diffusing particle, was characterized using dynamic light scattering (Nanosizer S90, Malvern Instruments). The zeta potential and isoelectric point (IEP) are primary indicators for describing the surface charge and stability of MWCNT suspension, and were determined using a zeta potential device (Zeta ZS, Malvern Instruments) with the pH value of the suspension adjusted from 2 to 7 using HCl.

### Pulmonary function testing

Thirty days following instillation of MWCNTs, all mice were anesthetized, tracheostomized, and placed on the FlexiVent system (SCIREQ, Montreal, QC, Canada) for forced oscillatory measurements. Mice were anesthetized with tribromoethanol (TBE) (400 mg/kg) and paralyzed with pancuronium bromide (1 mg/kg) to prevent spontaneous breathing. Mice were ventilated with a tidal volume of 10 mL/kg at a frequency of 150 breaths/min and a positive end expiratory pressure of 3 cm H_2_O to prevent alveolar collapse. Total lung capacity (TLC), Snapshot, Quickprime-3, and pressure-volume (PV) loops with constant increasing pressure (PVr-P) were consecutively performed using the Flexivent system. A TLC perturbation maximally inflates the lungs to a standard pressure of 30 cm H_2_O followed by a breath hold of typically a few seconds to establish a consistent volume history. A snapshot perturbation maneuver uses a three-cycle sinusoidal wave of inspiration and expiration to measure total respiratory system resistance (R), dynamic compliance (C), and elastance (E). A Quickprime-3 perturbation, which produced a broadband frequency (0.5 to 19.75 Hz) over 3 seconds, measures Newtonian resistance which is a measure of central airway resistanc (Rn), inertance (I), tissue damping (G), tissue elastance (H) and hysteresivity (eta). PV loops were generated between 30 cm H_2_O to -30 cm H_2_O pressure to obtain vital capacity (A), the upper portion of the deflation PV curve (K), quasi-static compliance (Cst) and elastance (Est), and the area of PV loop (Area). All perturbations were performed until three acceptable measurements with coefficient of determination (COD) ≥ 0.9 were recorded in each individual subject.

### BAL and cell differential counts

After measuring pulmonary function parameters, the right lung of each mouse was lavaged *in situ *four times with a specific volume (26.25 mL/kg body weight) of ice-cold Hanks balanced salt solution (HBSS). The first aliquot of bronchoalveolar lavage fluid (BALF) was collected separately for cytokine analysis, while aliquots 2-4 were pooled. All BALF was centrifuged at 1000 g for 10 min at 4°C. Total cells from all lavages were pooled and counted and 20,000 cells were centrifuged using a Cytospin IV (Shandon Scientific Ltd., Cheshire, UK) and stained with a three-step hematology stain (Richard Allan Scientific, Kalamazoo, MI, USA). Cell differential counts were determined by morphology with evaluation of 300 cells per slide.

### Lung histopathology

Unlavaged left lungs from C57BL/6 mice in each treatment group were inflated with 10% neutral buffered formalin fixative for 24-72 hrs. Lung tissue was then cut, processed, and embedded in paraffin, and 5 μM sections were mounted on slides. Sections were stained with hematoxylin and eosin (H&E) or Masson's trichrome stain to detect inflammatory, morphological changes and collagen deposition.

### Sircol collagen assay

Soluble collagen content within total lung was determined using the Sircol Collagen Assay (Biocolor, Belfast, UK). Lavaged right lung was collected and homogenized in 2 mL of RIPA buffer including protease inhibitors. Supernatants were collected from each sample after centrifugation at 10,000 g for 10 minutes at 4°C. Sircol dye reagent (1 mL) was added to 50 μL of each sample or varying concentrations of collagen standard to generate a calibration curve. Following a 30 min incubation period, samples were centrifuged at 10,000 g for 10 minutes. The collagen-dye precipitate was reconstituted in 1 mL of alkali reagent and absorbance was read at 540 nm and total collagen content was calculated from the standard curve.

### Mouse fibrosis PCR-array

Total RNA from left lung tissue of additional mice was isolated using a Qiagen RNeasy Mini Kit (Qiagen, Valencia, CA, USA) according to the manufacturer's recommendations. Total RNA (2.5 μg) was reverse-transcribed to cDNA using SABiosciences's RT^2 ^First Strand Kit (Qiagen, Frederick, MD, USA) and applied to Mouse Fibrosis PCR Arrays (Cat.# PAMM-120; 96-well format) following SABiosciences recommendations. Real-time PCR were performed in an Applied Biosystems StepOnePlus Real-Time PCR System (ABI, Foster City, CA, USA). Data were interpreted using SABiosciences' web-based PCR array analysis tool.

### qPCR

Total lung RNA was reverse transcribed using a QuantiTect reverse transcription kit (Qiagen). Quantitative real-time PCR was performed using QuantiTect primer assays and SYBR green master mix to verify expression of Ccl3, Ccl11, Mmp13, and IL-33 found in the mouse fibrosis PCR-array. An Applied Biosystems StepOnePlus Real-Time PCR System (ABI) was used to obtain cycle threshold (Ct) values for target and internal reference cDNA levels. Target cDNA levels were normalized to GAPDH, an internal reference, using the equation 2^-[ΔCt]^, where ΔCt is defined as Ct_target_- Ct_internal reference_. Values shown are the average of six independent experiments.

### ELISA and MMP13 Activity Assays

Chemokine (CCL3 and CCL11) and cytokine (IL-33) levels were quantified in BALF using DuoSet ELISA kits (R&D Systems, Minneapolis, MN) in accordance with the manufacturer's instructions. MMP13 activity assays were performed using SensoLyte^® ^MMP-13 Assay Kit (AnaSpec, San Jose, CA). BALF (50 μL) containing MMP13 was activated with 1 mM 4-aminophenylmercuric acetate (APMA) and the assay was performed according to the manufacturer's recommendations. Fluorescence, resulting from enzyme-mediated conversion of the fluorogenic substrates, was immediately measured by a Synergy™ HT Multi-Detection Microplate Reader (BioTek, Winooski, VT) using black, round-bottom 96-well plates (Corning, Corning, NY) and continuously recording data every 5 min for 60 min. The relative fluorescence unit (RFU) was calculated by subtracting fluorescence in the substrate control well from all experimental wells. The MMP13 activity of test samples was calculated as the initial reaction velocity (V_o_) in RFU/min.

### Statistical Analyses

All data are presented as means ± SEM and were analyzed by one-way ANOVA, with differences between groups assessed using Bonferroni *post hoc *tests. Graphs and analysis were performed using GraphPad Prism 5 software (GraphPad, San Diego, CA). Differences were considered statistically significant at *p *< 0.05.

## Competing interests

The authors declare that they have no competing interests.

## Authors' contributions

XW and PK equally carried out the experimental design and drafted the manuscript. RP and AR performed the SEM, TEM, Raman spectroscopy and TGA analysis of the MWCNTs. PC and PCK determined the MWCNT surface area, pore size, IEP and zeta potential. DW and CW participated in design of the study and interpretation of the data and editing of the manuscript. JB conceived of the study, design and coordination of the experiments as well as writing of the manuscript. All authors read and approved of the final manuscript.
